# Hair Cortisol and Testosterone Concentrations in Relation to Maturity and Breeding Status of Male Feral Horses

**DOI:** 10.3390/ani13132129

**Published:** 2023-06-27

**Authors:** Sarah A. Medill, David M. Janz, Philip D. McLoughlin

**Affiliations:** 1 Department of Biology, University of Saskatchewan, Saskatoon, SK S7N 5C8, Canada; philip.mcloughlin@usask.ca; 2 Department of Veterinary Biomedical Sciences, University of Saskatchewan, Saskatoon, SK S7N 5B4, Canada; david.janz@usask.ca

**Keywords:** cortisol, testosterone, non-invasive sampling, hair, dominance, feral horse, *Equus ferus caballus*

## Abstract

**Simple Summary:**

Hormone concentrations derived from hair can inform us of many aspects of an individual’s life, such as their reproductive status or social position. Cortisol is often associated with physiological or even psychological stress, while testosterone is strongly associated with male development and reproductive success. This study investigated cortisol and testosterone concentrations of tail hair collected from feral horses living in a naturally established social structure. We compare values of hair derived cortisol (*n* = 153) and testosterone (*n* = 48) to an individual’s social position as either an Immature male (2–4 years old), physically mature but non-reproductive male (Bachelor), reproductive dominant male (Stallion), or subordinate harem-associated male (tag). Immature males had significantly lower hair cortisol concentrations than adults. Hair testosterone concentrations were significantly higher in Stallions than males in the other social positions when age is accounted for. Bachelors exhibited a positive correlation between the two hormone levels, while among Stallions the association was negative. These findings suggest that patterns in hair hormone concentrations reflect known physiological interactions between cortisol and testosterone in relation to age, dominance, and reproduction, that have been established in the literature.

**Abstract:**

Steroid-hormone concentrations from non-invasively obtained biomarkers, like hair, can provide a representation of circulating hormones diffused over relatively long time periods (e.g., weeks or months). The hormone cortisol is often associated with physiological or even psychological stress, while testosterone is strongly associated with male development and reproductive success. Increasingly, studies are using hormone levels derived from hair to make inferences among both domestic animals and wildlife. For horses, all previous hair hormone analysis has been done on companion or working animals. We evaluated the levels of hair cortisol (*n* = 153) and testosterone (*n* = 48) from 136 feral horses living on Sable Island, Canada that have been part of a long-term individual-based study since 2008. This population has been undisturbed and unmanaged for over 50 years, and exhibits the natural social organization for horses, harem defense polygyny. Hair samples were collected in mid to late summer and the segment analyzed corresponds with hair grown during, and following, the peak of the reproductive season. Social position was determined based on the male’s role as either a dominant breeding Stallion (Stallion), a non-breeding subordinate male (tag), adult Bachelor (5 years old or older), or Immature male (2–4 years of age). While there was no difference in hair-cortisol concentration among any class of adult males (i.e., Stallion, tag, or Bachelor), Immature males had significantly lower hair cortisol concentrations than the other groups (*p* = 0.001). Hair testosterone levels among the four social positions were significantly higher among Stallions (*p* = 0.04). Hair testosterone concentration was also significantly related to the probability of a male being either a Bachelor or Stallion and was the only variable remaining in AIC_c_ model selection (*p* = 0.016, AIC_c_ = 32.3, Null AIC_c_ = 38.8). While not a significant relationship, Stallions had a negative correlation between hair cortisol concentrations and testosterone (R^2^ = −0.20, *p* = 0.383), and Bachelors, conversely, had a positive association (R^2^ = 0.43, *p* = 0.246). Our observations of hormone concentrations in relation to physiological, social, or reproductive parameters in this population suggest trends that are similar to what has been established using blood or other matrices.

## 1. Introduction

Moderating androgen hormones such as testosterone and glucocorticoid hormones (e.g., cortisol or corticosterone) are likely to mirror how individuals interact with their environment, including conspecifics in a social setting [1,2,3,4]. In mammals, particularly where social hierarchies exist, the males with the highest testosterone levels tend to maintain more dominant positions, while subordinates have lower levels of this hormone [1,5]. The role of glucocorticoids in relation to social position is, however, less clear, with high levels potentially found in both dominant and subordinate individuals depending on the type and stability of the social structure [5,6,7]. In general, glucocorticoid levels rise when there is an actual or perceived (e.g., psychological stress) need for the mobilization of stored energy [8,9,10], which may be divorced from social or reproductive status.

The hypothalamus–pituitary–gonadal axis (HPG; regulating testosterone production) and the hypothalamus–pituitary–adrenal axis (HPA; regulating glucocorticoid production) do not operate as independent systems and can be activated by some of the same physiological and psychological stimuli [11,12]. Additionally, the two axes can have an inhibitory effect on one another and on other systems in the body. Testosterone is known to have an inhibitory effect on HPA function [13,14,15], while it has also been shown that basal cortisol levels can influence the production of testosterone [14,16,17,18,19,20,21]. Furthermore, responsiveness of the HPA axis to stress during reproductive periods may be linked to life history and social structures [4,16].

Like glucocorticoids, testosterone levels are modified by several physical and psychological processes [22,23]. Testosterone increases in preparation for contests, and generally, the winners of these contests proceed to generate even greater amounts of testosterone [11,24,25,26,27,28,29,30]. Circulating testosterone levels also rise with an increase in sexual behavior, particularly in mammals [1,29,31], including horses [32,33,34], and with behaviors related to mate acquisition [31,35], and in anticipation of the breeding season [10]. Quantifying testosterone and glucocorticoid concentrations in the context of sociality may help to understand related properties such as breeding success or social position.

The polygynous harem-defense social structure exhibited by feral horses provides an opportunity to investigate the relationship of testosterone and cortisol. In the wild, successful males acquire and defend their access to one or more breeding-age females through physical contests [36,37], and they maintain these associations year-round. Males that are unsuccessful in acquiring or retaining access to females will often form all-male groups or remain solitary. As an alternative male strategy, sometimes a subordinate male, referred to here as a ‘tag’ male, will associate with a particular band (group composed of the dominant male, mature females, and their related offspring) and contribute towards the band’s defense. Tag males may gain limited or opportunistic access to females of the group, and have been shown to have higher reproductive success than bachelors [36,37,38,39]. Male horses may produce semen as young as two years old; however, males less than 5 years old are rarely seen defending or breeding with females in the wild [37].

Traditional matrices (such as blood serum, saliva, urine, or feces) reflect only the most current (minutes to hours) circulating hormone levels [40], and often require capture to obtain, which could influence the sample and disrupt natural social organization. Steroid hormones recovered from hair arise from passive diffusion of circulating free (unbound) hormone integrated during the hair growth and provide a representation of long-term circulating concentrations [41,42,43]. Applications of hair hormone analysis investigating simultaneous HPA and HPG activity has been applied to determine age and reproductive class to male grizzly bears [44]. In horses, a similar investigation looked at seasonal variation in hormone levels using hair samples [45]. The feral horses of Sable Island, Nova Scotia, Canada, present a model system in which to study non-invasively collected hair samples for hormone concentration in relation to social position of the males. The roughly 500 horses on Sable Island exhibit a naturally organized social system in the absence of predators or other outside sources of disturbances, including human interference, which could influence an individual’s hormone levels [46]. The population is also the subject of a detailed, individual-based study of ecology (since 2008), in which age and breeding status are determined for all males during repeated summer surveys (July–August).

Based on previous literature regarding testosterone levels and the winner effect [25,27,29,30,34], and in relation to courtship opportunities [1,29,31,32,33], we predicted that dominant band stallions should have higher levels of testosterone than bachelors, as they monopolize the courtship and mating opportunities and obtain (or maintain) their position through winning contests. Additionally, we predicted that males with high levels of cortisol would be more likely to have lower levels of testosterone due to the suppressive influence of HPA activity on HPG function, and conversely, that males with high hair testosterone levels will have lower hair cortisol based on the suppressive effects of HPG activity on the HPA axis. Our objective was to present an analysis of matching hair hormone concentrations with known social position for our study population and to further the use of non-invasively collected biomarkers, like hair, as part of sociobiological research for wild populations.

## 2. Materials and Methods

### 2.1. Population Description and Social Positions

The feral horses living on Sable Island (Nova Scotia, Canada) have existed since the mid-1700s, and have established a wild-type social structure, and have not experienced human interference (e.g., additions or removal of individuals, or disruption to social groups) since at least the 1960s, when their protected status was formalized [47]. This population is surveyed annually in summer (July–August) by researchers at the University of Saskatchewan (starting in 2008) to identify individuals, determine fates of life history, and test hypotheses of behavior and evolutionary ecology.

During the survey, individuals are photographed thoroughly, and identification is carried out through manual recognition of natural markings, scars, whorls, or any other distinguishing characteristics. Age (birth year) of the individual is based on their first sighting as a foal or as a yearling, which are distinguished from other ages based on size and mane length. While the study formally started in 2008, photos taken during a 2007 reconnaissance allowed us to age a portion of the 2006 cohort. However, this does leave us with an overlap of horses known to be age 6 and a group of horses of unknown age that may be 6 years old or older for this study.

Male horses are categorized into one of four social positions based on age and breeding status: Immature, Bachelor, Stallion, or Tag. Males aged 2–4 years are considered Immature, because while they can produce semen, they do not obtain access to females, and are still undergoing physical development and growth. We consider males 5 years and older as adults, as this is the first age at which we have seen males acquire and retain a harem in this population (this study). Bachelors are males ≥5 years old that are not associated with any females. The Stallion group consists of the dominant, or sole, adult breeding male that defends a harem, while any subordinate males in these breeding groups ≥5 years old are classified as Tag males. Determination of the dominant versus subordinate male in a group is easily distinguished by their proximity and interactions with females; dominant males are often closer and have more interactions with females, while Tag males are generally on the periphery of the group and usually first to engage with Bachelors or other band Stallions [37,38,48]. In this population, the prevalence of multi-male bands was 11% (2011) and 14% (2012) of all bands in each year [46].

### 2.2. Sample Collection and Processing

In 2011 and 2012, rooted tail hairs were originally collected for microsatellite DNA analyses and for hormone analysis (this study) under our institutional animal care permit (University of Saskatchewan UCACS #20090032). Hair samples were collected opportunistically from unrestrained individuals either by deploying purposely built hair snags (with observed use to identify the individual) or by plucking directly. Nitrile gloves were worn during collection and samples were placed into envelopes and stored in the dark at ambient temperature until processing.

Only hairs with the roots visibly attached were used in this analysis. We removed 4 mm of the root end to retain for DNA analysis and used the next 5 cm segment to evaluate hormone concentrations. Tail growth rates of domestically kept horses have been reported as being between 0.066 and 0.081 cm/day [49,50,51,52]. Based on these published rates of growth, the 5 cm segment of tail hair would represent the time between 67–82 days prior to sample collection, excluding the most recent 5–6 days associated with the 4 mm portion of hair root removed. Hair samples were collected between July 23rd and August 22nd in both 2011 (*n* = 26) and 2012 (*n* = 127). We therefore captured the hormone profile within the hair shaft corresponding to a period of time that would reflect peak parturition (May) and breeding activity, which continues into June and July [36,37].

We analyzed the hair samples following procedures described in Macbeth et al. [53], validated for feral horse hair. Please see Medill et al. [54] for additional details on sample processing and modifications relating to the use of hair segments. Methanol washes to remove exogenous sources of hormones and contaminants (e.g., from sweat or feces) were performed after the removal of the root end but before the 5 cm segment was isolated from the full hair shaft. Up to 25 mg of powdered hair sample was analyzed by extracting hormones with HPLC-grade methanol over 24 h and reconstituted using the phosphate buffer provided with the appropriate kit. Hair cortisol concentrations (HCC) and hair testosterone concentrations (HTC) were quantified using commercially available enzyme-linked immunosorbent assay kits (Cortisol–EA-65 Cortisol EIA kit, Oxford Biomedical, Oxford, MI, USA; Testosterone–ADI-900-065 Testosterone EIA kit, Enzo Life Sciences, Plymouth Meeting, PA, USA).

### 2.3. Validation of the Enzyme Immunoassays

Using feral horse hair extracts, intra-assay percent coefficient of variation (%CV) for cortisol was 6.8% (*n* = 6) and for testosterone it was 2.5 % (*n* = 6), and inter-assay %CV for cortisol was 8.3% (*n* = 12) and for testosterone it was 5.7% (*n* = 12). The limit of detection for cortisol was 0.02 ng/mL, and parallelism between the kit standard curve and serially diluted feral horse hair extracts was observed (R^2^ = 0.997, *p* < 0.001; see [53] for methodological details). The limit of detection for testosterone was 6.71 pg/mL, and parallelism between the kit standard curve and serially diluted feral horse hair extracts was observed (R^2^ = 0.983, *p* < 0.001).

### 2.4. Statistical Analyses

All statistical analyses and graphics were performed with the software package R version 4.2.2 [55]. The Shapiro–Wilk normality test was performed on both HCC and HTC data. Hair cortisol concentrations were skewed to the right, but log10 transformation produced a normal distribution for statistical analysis (W = 0.99, *p* = 0.576) and this transformed value was used in all further analysis. Hair testosterone concentrations were normally distributed (Shapiro–Wilk test, *W* = 0.97, *p* = 0.168) and did not require transformation.

Of the 153 cortisol samples, 17 were from individuals that were sampled in both 2011 and 2012 for a total of 136 unique individuals. A Linear Mixed Effect Model (nlme; [56]) was used to look at the relationship of HCC to both social position and year while using horse ID as a random factor to account for the repeated measures from the 17 individuals sampled both years.

Only a subset of the individuals had sufficient ground hair sample remaining to test for testosterone (*n* = 48). Of these, only 2 samples were from 2011, and the remainder came from individuals in 2012; none were repeated samples from the same individual, and due to the small number from 2011, year was dropped as a factor of interest, as it would have little influence nor statistical robustness. A linear mixed effect model was used to look at HTC in relation to social position; age was included as a random factor, since our classifications are age dependent, but the different ages are also unevenly represented in our sample.

We restrict our last analysis to Bachelors and Stallions to focus only on males that have attained full maturity; we also exclude the four Tag males, as they have an intermediary position, and their low number of samples does not make for robust statistics. Bachelor and Stallion are then treated as a binary variable in a generalized linear mixed effect model and use AIC_c_ model selection criterion [57]. The full model evaluated included fixed terms: Age, HCC, HTC, and the interaction term HCC*HTC. The glm and resulting figures used the following packages in R: lme4 [58], MuMin [59], tidyverse [60], lmerTest [61], pROC [62], emmeans: [63].

## 3. Results

### 3.1. Hair Cortisol Concentrations

Using a mixed effects model to look at HCC in relation to social position and year with the random factor of horse ID, we observed significant differences in male HCC between the years, with 2011 (*n* = 26, range = 0.46–4.38 pg/mg, mean = 1.55 pg/mg) having lower hair cortisol levels than 2012 (*n* = 127, range = 0.37–9.50 pg/mg, mean = 2.28 pg/mg), *p* = 0.005, Table 1). Cortisol levels observed in Immature males (*n* = 44, range = 0.46–7.10 pg/mg, mean = 1.57 pg/mg) were also significantly lower than Stallions (*n* = 70, range= 0.37–9.50 pg/mg, mean = 2.30 pg/mg), subordinate Tag males (*n* = 11, range = 0.66–6.19 pg/mg, mean = 2.43 pg/mg), or adult Bachelors (*n* = 28, range = 0.67–6.40 pg/mg, mean = 2.63 pg/mg; Table 1).

### 3.2. Hair Testosterone Concentrations

**Figure 1:**
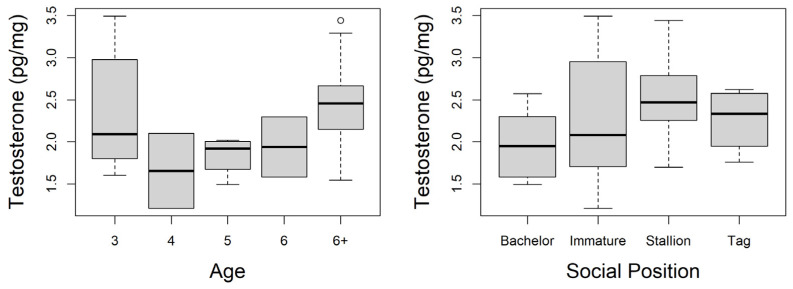
Hair testosterone concentrations from male feral horses based on their age (left) and social position (right). Sample sizes for Age: 3 (*n* = 12), 4 (*n* = 2), 5 (*n* = 4), 6 (*n* = 2), and 6+ (*n*=28). A significant difference between Stallions and other social positions was observed (age included as random factor in model; *p* = 0.04). Adult non-breeding males (Bachelor, *n* = 9), Immature males aged 3-4 years old (*n* = 14), breeding males in a dominant role (Stallions, *n* = 21); and subordinate males with limited breeding opportunities (Tag, *n* = 4).

HTC was compared to the age of the individuals (Figure 1). No difference in HTC was detected (Anova; *p* = 0.071); however, the reader should take note of the uneven and low sample sizes for some age groups and recognize that this does not allow for rigorous or reliable testing for differences between age groups. The following is the sample size, range, and mean for each age group: 3 year olds (*n* = 12, range = 1.60–3.49 pg/mg, mean = 2.35 pg/mg), 4 year olds (*n* = 2, range = 1.21–2.10 pg/mg, mean = 1.66 pg/mg), 5-year olds (*n* = 4, range = 1.50–2.02 pg/mg, mean = 1.84 pg/mg), 6 year olds (*n* = 2, range = 1.59–2.30 pg/mg, mean = 1.94 pg/mg), and horses 6 years and older (*n* = 28, range = 1.55–3.44 pg/mg, mean = 2.43 pg/mg). These uneven sample sizes among the lower ages was one of the reasons we focused on social position; horses that are 3 and 4 years old compose the Immature group. The Bachelor group consists of three 5-year-olds, one 6-year-old, and five horses in the 6 and older age group. The Stallion group has one 5-year-old, one 6-year-old, and 19 individuals identified as 6 years or older. Tag males were all from the 6 years and older age group.

Using a linear mixed effect model to look at HTC by social position, with age included as a random factor, we determined that Stallions have a significantly higher HTC than the other groups (for Stallions: *p* = 0.04). The sample size, range, and mean HTC of the four social positions are as follows: Immature males (*n* = 14, range = 1.21–3.50 pg/mg, mean = 2.25 pg/mg), Bachelors (*n* = 9, range = 1.50–2.57 pg/mg, mean = 1.97 pg/mg), Stallions (*n* = 21, range = 1.70–3.44 pg/mg, mean = 2.51 pg/mg), and Tag males (*n* = 4, range = 1.76–2.62 pg/mg, mean = 2.26 pg/mg).

**Figure 2:**
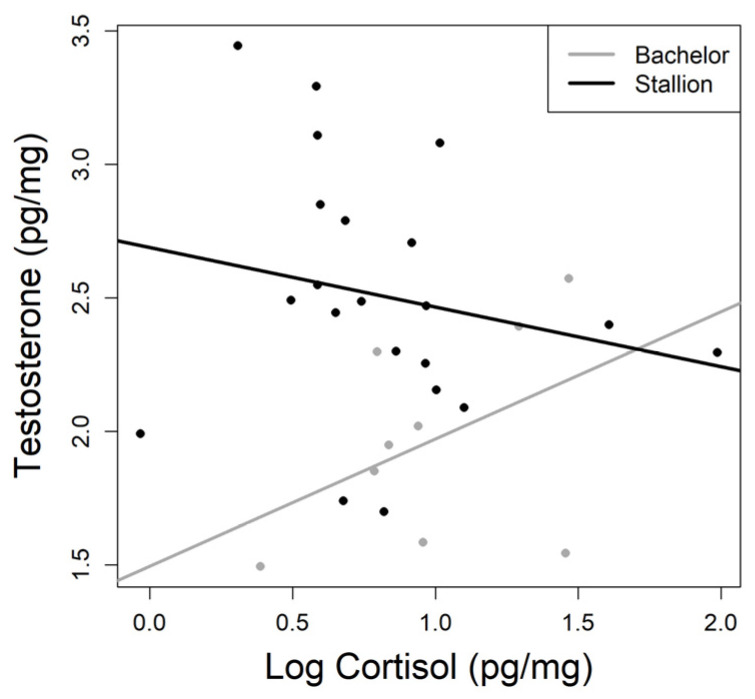
Hair testosterone concentrations for adult, non-breeding, male horses (Bachelor, grey; *n* = 9, R^2^ = 0.43, *p* = 0.246) and dominant breeding males (Stallion, black; *n* = 21, R^2^= −0.20, *p* = 0.383) in relation to hair cortisol concentrations. Interaction term is not statistically significant.

When looking at the figure of HTC and HCC among only Bachelors and Stallions (Figure 2), we observed a potential interaction between hair cortisol and testosterone hormone levels. For Bachelors, as HCC increases, we see an increase in HTC (R^2^ = 0.43, *p* = 0.246). Meanwhile, for Stallions, we observed a negative relationship (R^2^ = −0.20, *p* = 0.383), and while neither of these relationships were significant, we were interested in the difference in response direction for the two groups.

**Figure 3:**
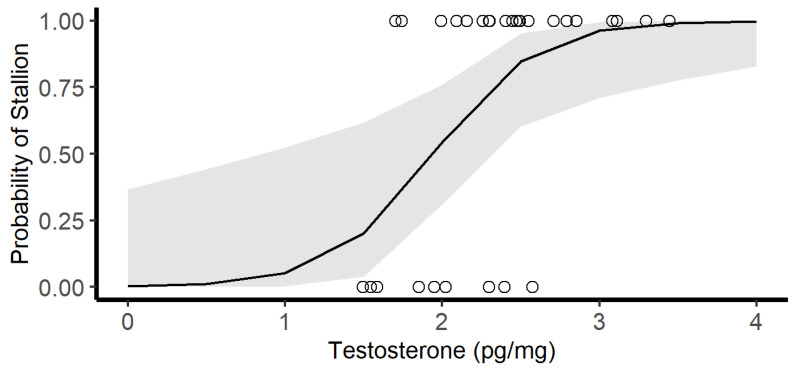
Probability of being Bachelor (0) versus Stallion (1) can be partially predicted by concentration of hair testosterone, shaded area represents 95% confidence interval (glm: *p* = 0.016, AIC_c_ = 32.3, Null AIC_c_ = 38.8).

Our investigation of the full generalized linear model to explain Bachelor vs. Stallion position included the fixed factors age, HCC, HTC, and the interaction term between HCC and HTC; no levels, variables, or the interaction term in the full model were significant (Table 2). The model with the lowest AIC_c_ includes only the fixed factor of HTC, which was significantly related to probability of Stallion vs. Bachelor (*p* = 0.016, AIC_c_ = 32.3, Figure 3). The next best model included HCC with HTC though it is interesting to note that the poorest-fitting model is HCC on its own. The interaction term HCC*HTC is in the third top model (∆AIC_c_ = 3.61). As a fixed effect, age is less influential; however, as we are restricting this to adult Bachelors and Stallions only, age here is only composed of three groups (Age 5, 6, and 6+), which are very unevenly weighted.

## 4. Discussion

Non-invasively collected biomarkers, such as hair, that are capable of recording hormone levels over long periods of time are gaining interest in both wildlife and domestic animal endocrinology. The use of hair in these analyses also has some other practical benefits, such as relatively simple collection and storage requirements [40,44]. While individual-based, longitudinal studies are rare among wild populations, it is evident from our work that hair hormone analysis could be applied to monitor endocrine state as individuals’ transition between pubescence and into reproductive stages of their lives.

No significant differences were found in cortisol levels between the adult social classes (Bachelor, Stallion, or Tag); however, immature males (3–4 years old) had lower hair cortisol concentrations. Low cortisol concentrations among juvenile or late-pubescent males have been detected in other species and have been suggested to be linked to the inhibitory influence of testosterone on the HPA axis acting prior to the onset of stress induced by reproductive competition [64,65]. We did see high testosterone concentrations among individuals at 3 years old, so there may be some support for a similar interaction occurring within feral horses. The lack of significant difference in cortisol between adult males occupying different social positions is not surprising either given the role of this hormone to assist in meeting a variety of energetic demands. Winning males (i.e., dominant band stallions) have high energy demands related to their reproductive roles but perhaps do not experience the rise in cortisol related to the social defeat that subordinate males and bachelors may experience [66,67]. It was informative that the model for position as bachelor or stallion had the worst fit when HCC was the only variable included.

As predicted, hair testosterone concentrations were generally greater in dominant breeding band stallions compared to non-breeding adult males (bachelors). Males that had established themselves as dominant band stallions would have done so through successfully winning contests or by continuing to defend their social position against other males, whereas bachelor males would have either lost, or not attempted, these contests, and would thus have had little or no access to reproductive opportunities [37,38,39]. Although the sample size was too low to draw conclusions on the hair hormone profiles of males choosing an alternative mating strategy (tag males, *n* = 4), it is interesting to note that hormone profiles of these males fell into an intermediate range between non-breeding bachelors and dominant breeding males. The lower number of samples in this study from tag males is also a reflection of the fact that it is not a frequent strategy employed by males in this population [46].

As is well documented for cortisol [8,40], circulating testosterone levels can change rapidly in response to sexual or social triggers such as the winning or losing of contests [11,24,25,28,30]. In many species, courtship behaviors or intercourse leads to an increase in testosterone production [29,31]. The combination of repeated experiences that dominant band stallions have, both defending their harem and in terms of their reproductive activities, gives plausible support to our observations of higher testosterone concentration in the hair of dominant band stallions. Stallions defending access to females from a group of bachelors showed a rapid increase in serum testosterone at the start of their tenure and a rapid decrease when they were removed from that position [34]. Similarly, bison that were guarding potential future mates during the reproductive season were also more likely to have higher testosterone levels than males that were not [68]. Additionally, through non- or minimal-contact posturing, band stallions can bring an early end to approaches by subordinates and bachelors without escalating to injurious fighting [37]. It has been shown in other animals that even the anticipation of a contest or fight can result in elevated testosterone production [26,69], so it is possible that a stallion defending a harem through posturing behavior could also be raising systemic testosterone levels.

The interaction between cortisol and testosterone levels on an individual’s performance has led to the development of the dual-hormone hypothesis [19,70]. This hypothesis suggests that the effectiveness of testosterone is related to the level of glucocorticoids. Glucocorticoids have been shown to have a moderating effect on androgen-based behavioral or phenotypic traits which can in turn influence sexual selection [21] and conspecific interactions [71]. When glucocorticoids are low, the enhancing effects of testosterone are more pronounced, but when glucocorticoid levels are high, the relationships between high testosterone and dominance or aggressive behaviors tends to break down [19,19,70,72]. While the correlations between HTC and HCC for bachelors and stallions were not significant, there was a difference in the direction of the relationships that may have a biological explanation. Our data suggest that, among adult males with low HCC levels, there were clear differences in social position with HTC for stallions being much higher than for non-breeding bachelors. Interestingly, at higher values of HCC the difference in testosterone between stallions and bachelors was reduced. This could be reflecting the inhibitory effect of cortisol upon testosterone production identified in other organisms [14,16,17,18,19,20,21]. Comparing this from a different point of view, the bachelors with the lowest cortisol also had the lowest testosterone, while those with higher cortisol levels had the highest testosterone levels, which may suggest that these individuals are perhaps more actively competing for mating opportunities and are perhaps investing more energy in these behaviors; unfortunately, we lack the concurrent behavioral data to test these hypotheses.

Although the use of hair for hormone analysis may be attractive for understanding endocrine states in wildlife there are still some problematic aspects to its use. Variation in hair growth rates between individuals [52] or the potential for a positive influence of testosterone on hair growth [50] could influence the exact period of time represented in the hair segment, and it is unknown how variations in growth rate may affect the incorporation of hormones into the hair. Mane and tail hair of horses grow nearly continuously, with a large proportion of the hairs being in active growth at any given time [50,52,73]. Although some degree of variation in hair growth is expected among our individuals, the segment used in this analysis likely remains representative of the prolonged breeding season of feral horses which extends into June and July [36,37] and we are confident that the observed hair hormone concentrations reflect differences in the circulating levels of these hormones [74,75].

This research is also limited to investigating the hormone patterns from a single hair collection in mid-late summer. For seasonal breeders, like horses, levels of testosterone and cortisol can vary throughout the year [23,32]. Seasonality in cortisol [45,76] and testosterone [45] have both been found in domestically kept horses. Year of collection was included in this analysis, as it represents a biological factor that broadly impacts the population. Most samples were collected in 2012, a year that has been identified as a drought year [77]. The impact of resource stress and resulting shifts in landscape use could be a reason we see a significant rise in cortisol levels in 2012 [54]. Further evaluation of hair cortisol concentrations in association with year and along with other social and biological attributes in Sable Island horses, including females, will be presented in a separate publication (in preparation). Different results may be expected when looking at hair hormone patterns in domestic horses compared to feral populations experiencing a full repertoire of natural social and reproductive behaviors and seasonal resource limitations [54,77,78].

## 5. Conclusions

Our observations suggest that the complicated relationship between cortisol, testosterone, and the maturity and breeding status of horses is reflected in hair hormone concentrations. Testosterone concentrations derived from hair revealed an anticipated pattern of males that had won breeding contests and maintained an active role in reproduction having higher testosterone levels than males that had failed to obtain or maintain breeding opportunities. Among Immature males, we saw patterns of low cortisol and the potential to have higher levels of testosterone. Considering that the effectiveness of testosterone in developing the phenotypes or behaviors related to sexual selection can be dependent on cortisol levels, it is possible that a balance of these (and other hormones) may be critical for achieving later reproductive success in feral horses. Despite the non-significant interaction term, our data hint that an opposite relationship between cortisol and testosterone is present for breeding vs. non-breeding adults. Hair hormone concentrations represent long-term levels of circulating hormones making them an ideal matrix to investigate this pattern in feral populations. Future studies could be improved with the addition of more detailed behavioral data (e.g., contests) and larger sample sizes. We also encourage the development of additional baseline studies in feral horse populations matching hormone concentrations with hierarchical rank, and investigate across seasons, to further the use of non-invasive biomarkers, such as hair, as part of sociobiological and ecological research in the wild.

## Figures and Tables

**Table 1:** Estimate of fixed effects for social position and year on log-transformed hair cortisol concentration (horse ID as random variable).

Parameter	Estimate	se	df	*t*	*p*-Value	Lower 95% CI	Upper 95% CI
Intercept	0.5306	0.143	134	3.716	<0.001	0.248	0.8129
Social Position: Bachelor	0	0	-	-	-	-	-
Social Position: Immature	−0.550	0137	14	−4.025	0.001	−0.843	-0.257
Social Position: Stallion	−0.124	0.128	134	−0.968	0.335	−0.378	0.129
Social Position: Tag	−0.173	0.1866	14	−0.929	0.369	−0.574	0.227
Year: 2011	0	0	-	-	-	-	-
Year: 2012	0.3340	0.100	14	3.333	0.005	0.119	0.549

**Table 2:** Models of social position as either Bachelor or Stallion based on the fixed variables age, hair cortisol concentration (HCC), hair testosterone concentration (HTC) or interaction of the two variables HCC * HTC. Asterisks indicate inclusion of a factor variable in the model.

Intercept	Age	HCC	HTC	HCC * HTC	df	AIC_c_	∆AIC_c_	logLik
−5.99			3.08		2	32.3	0.00	−13.93
−5.44		−1.33	3.41		3	33.4	1.14	−12.26
−8.73		2.50	4.94	-1.76	4	35.9	3.61	−13.15
−5.81	*	−2.66	3.17		5	36.1	3.82	−11.81
−5.99	*		2.62		4	36.6	4.27	−13.48
−0.06	*	−2.36			4	38.0	5.66	−14.18
−1.10	*				3	38.8	6.46	−15.92
0.85					1	38.8	6.50	−18.33
−4.18	*	−4.61	2.38	0.86	6	39.2	6.94	−11.79
1.84		−1.10			2	39.9	7.59	−17.72

## Data Availability

The data presented in this study are available on request from the corresponding author.
